# Adjunctive Nutraceutical Therapies for COVID-19

**DOI:** 10.3390/ijms22041963

**Published:** 2021-02-16

**Authors:** Lalita Subedi, Stephanie Tchen, Bhakta Prasad Gaire, Bingren Hu, Kurt Hu

**Affiliations:** 1 School of Medicine, University of Maryland, Baltimore, MD 21201, USA; lsubedi@som.umaryland.edu (L.S.); bgaire@som.umaryland.edu (B.P.G.); bhu@som.umaryland.edu (B.H.); 2 Froedtert Hospital, Milwaukee, WI 53226, USA; stephanie.tchen@froedtert.com; 3 Department of Medicine, Division of Pulmonary and Critical Care Medicine, Medical College of Wisconsin, 8701 Watertown Plank Road, Milwaukee, WI 53226, USA

**Keywords:** novel coronavirus, pandemic, viral infection, nutraceuticals, antiviral therapy

## Abstract

The novel coronavirus, severe acute respiratory syndrome coronavirus 2 (SARS-CoV-2/COVID-19), is a worldwide pandemic, as declared by the World Health Organization (WHO). It is a respiratory virus that infects people of all ages. Although it may present with mild to no symptoms in most patients, those who are older, immunocompromised, or with multiple comorbidities may present with severe and life-threatening infections. Throughout history, nutraceuticals, such as a variety of phytochemicals from medicinal plants and dietary supplements, have been used as adjunct therapies for many disease conditions, including viral infections. Appropriate use of these adjunct therapies with antiviral proprieties may be beneficial in the treatment and/or prophylaxis of COVID-19. In this review, we provide a comprehensive summary of nutraceuticals, such as vitamins C, D, E, zinc, melatonin, and other phytochemicals and function foods. These nutraceuticals may have potential therapeutic efficacies in fighting the threat of the SARS-CoV-2/COVID-19 pandemic.

## 1. Background

Acute respiratory syndrome coronavirus 2, also known as the novel coronavirus (SARS-CoV-2/COVID-19), was first identified in December 2019 [1]. Since then, it has rapidly spread across the globe to approximately all countries and regions [2]. Therefore, in early 2020, the World Health Organization (WHO) declared COVID-19 a pandemic. Two recent vaccines have received United States Food and Drug Administration (FDA) emergency use approval based on promising clinical data against this viral infection [3]. Although vaccination is a step in the right direction, it may not provide complete protection against the infection; therefore, it will still be necessary to develop effective therapies against COVID-19.

COVID-19 is an enveloped, positive-sense, single-stranded ribonucleic acid (RNA) virus from the coronavirus family [4]. It can be transmitted from person-to-person by aerosolized droplet particles. Clinical presentation of the infection ranges from asymptomatic to mild upper respiratory tract infections to severe multi-organ failure and death [5,6]. The specific pathophysiology behind why COVID-19 produces such a broad spectrum of disease remains unknown; however, an aggravated inflammatory cascade has been implicated in the most severe of syndromes [7]. To date, a combination of therapies including anti-inflammatory, antiviral, and other medications with unique pharmaceutical properties have been evaluated for use in the treatment and/or prevention of COVID-19 [8]. Treatment regimens for viruses that share structural and genetic similarities, such as the Severe Acute Respiratory Syndrome (SARS-CoV-1) virus from 2003 and the Middle East Respiratory Syndrome (MERS-CoV) virus from 2012, have also been assessed [7,9]. Currently though, there remain limited medications that are approved for these indications by the FDA.

As modern drug discovery and development have stemmed from the application and evaluation of plants and natural products, nutraceuticals remain an important source of medicinal agents that may lead to novel treatment strategies [10]. Nutraceuticals include active phytochemicals isolated from plants, dietary supplements, and functional foods with medicinal properties [11]. Adjunctive use of these agents may also prove to be beneficial in mitigating the manifestations of COVID-19. These nutraceuticals include “immune boosting” foods and nutrients such as zinc, vitamins, garlic, turmeric, ginger, selenium, etc. (reviewed in [12,13]). In this review, we provide a brief overview of some of the conventional COVID-19 therapies being evaluated, as well as the potential therapeutic properties of nutraceuticals in the treatment and/or prophylaxis of COVID-19.

## 2. Methods

Separate PubMed and EMBASE searches were conducted to find relevant articles and studies related to the use of nutraceuticals in COVID-19. Keywords such as “coronavirus”, “SARS-CoV-2”, “COVID-19”, “phytochemicals”, “nutraceuticals”, “COVID-19 pandemic”, “dietary supplements”, and “functional foods” were used. Articles were excluded if they were not available in English. All articles were then complied and reviewed by three individuals (LS, ST, BPG) for relevancy and inclusion in this review.

## 3. Proposed Conventional Treatment Strategies for COVID-19

Several FDA approved medications are currently being evaluated for use in the treatment and prevention of COVID-19 (Table 1). These include the off-label use of medications such as azithromycin, hydroxychloroquine, tocilizumab, remdesivir, dipyridamole, and baricitinib. In May 2020, an emergency use authorization was issued by the FDA for the use of remdesivir in the treatment of suspected or laboratory confirmed COVID-19 in those with severe disease [3]. A study by Beigel et al. found that remdesivir reduced the time of recovery from a median of 15 days to 11 days in a randomized, placebo-controlled trial, which led to its FDA approval. However, the effect on the recovery time was not present in patients with mild-to-moderate disease or patients needing mechanical ventilation or extracorporeal membrane oxygenation. Remdesivir should be reserved for patients who are early in their disease course on supplemental oxygen but not yet intubated [14].

In June 2020, the National Institutes of Health (NIH) also released guidelines regarding the use of steroids in COVID-19 [28]. Initially, based on negative observational studies, glucocorticoids were vastly avoided in this patient population; however, preliminary results from an unpublished study from the United Kingdom found that dexamethasone at a dose of 6 mg/day for up to 10 days decreased 28-day mortality compared with standard of care (21.6% mortality in the intervention group vs. 24.6% in the control group; 95% CI 0.74–0.92, *p* < 0.001) [28,29] Moreover, a recent open-label multicenter trial found that the use of 20 mg of dexamethasone for five days followed by 10 mg of dexamethasone for five days increased ventilator-free days (6.6 ventilator-free days in the intervention group vs. 4.0 ventilator-free days in the control group; 95% CI 0.2–4.38, *p* = 0.01) [30]. This led to the recommendation from both the NIH and Infectious Disease Society of America (IDSA) to use dexamethasone in patients with COVID-19 that have increased oxygen requirements. Those requiring invasive mechanical ventilation showed the highest benefit, with a number needed to treat (NNT) of 8.5.

Convalescent plasma, or plasma collected from recovered COVID-19 patients, is another investigational treatment being used in patients with severe pneumonia from COVID-19 [31]. The plasma is thought to contain neutralizing antibiotics to facilitate clearance of the virus [32]. This strategy was also evaluated for use in previous viral outbreaks, such as the SARS-CoV-1 and MERS-CoV outbreaks. In May 2020, the FDA also released guidance regarding the use of investigational convalescent plasma in this patient population [33]. Recent trials involving convalescent plasma in the treatment of COVID-19 have had variable results. These variable results have led the IDSA to only recommend the use of convalescent plasma in the context of a clinical trial [34,35,36,37]. As an investigational agent, this approach should be used with caution as the safety of convalescent plasma therapy has not been fully studied, and its use may be associated with risks that could worsen the disease [38].

In November 2020, baricitinib, in combination with remdesivir, received an emergency use authorization by the FDA [25]. Baricitinib is an oral selective Janus kinase (JAK) 1 and 2 inhibitor. The JAK-STAT pathway has been implicated in the transferring of signals from cell membrane receptors to the nucleus and plays an essential role in the downstream effect of cytokines. A phase 3 study showed that the addition of baricitinib to remdesivir reduced the overall median recovery time by one day (7 days in baricitinib and remdesivir vs. 8 days in remdesivir alone; rate ratio for recovery 1.16; 95% CI 1.01–1.32, *p* = 0.03). Patients requiring either noninvasive ventilation or high-flow oxygen benefited the most, reducing the recovery time by 8 days (10 days vs. 18 days; rate ratio for recovery 1.51; 95% CI, 1.10–2.08). The investigators found fewer adverse events in the barcitinib group [25]. Addition of baricitinib to remdesivir in patients who require noninvasive ventilation and high-flow oxygen support should be highly considered. The reduced length to improvement is important and may reduce the congestions caused by the increased number of patients. An ongoing study is looking specifically at the effects of baricitinib in the COVID-19 population (NCT04421027).

In December 2020, bamlanivimab received emergency use authorization by the FDA [39]. Bamlanivimab, is a synthesized neutralizing antibody that targets a SARS-CoV-2 specific binding spike protein, preventing its binding to human angiotensin-converting enzyme 2 receptors. This is thought to produce similar effects to those of convalescent plasma. Interim analysis of the bamlanivimab phase 2 study showed a mean reduction in viral load at day 11 in treatment patients as compared to placebo (−0.22; CI 95% −0.60 to 0.15) when given within 3 days of testing positive and mild-to-moderate symptom onset. More importantly, it reduced the number of hospitalizations in the intervention group when compared to the placebo group (1.6% vs. 6.4%). Although initial data are promising, careful attention to patient selection is advised when administering bamlanivimab.

Several multinational pharmaceutical companies are working toward vaccine production against COVID-19. Fortunately, many of the vaccines are showing promising results based on the clinical trials. A few of these vaccine have shown great efficacy in preventing COVID-19 infection [3]. Both the Pfizer–BioNTech and the Moderna, Inc. vaccines have been approved by the FDA for administration under emergency use authorization [40,41]. In addition, the Oxford–AstraZeneca (AZD1222) vaccine is also in use for human patients in several European countries, although it is still undergoing the FDA approval process that is considering its potential to prevent COVID-19 infections [3].

Numerous other medications have also been evaluated for use in COVID-19; however, robust evidence promoting use of these agents is lacking. For example, tocilizumab has been evaluated for use in patients with moderate-to-severe disease and in those with worsening inflammatory parameters. Current data regarding its use are conflicting though, as several open label trials did not show improvement in their primary endpoints [42,43]. A recently completed randomized, double-blind, placebo-controlled trial analyzing the efficacy of tocilizumab on hospitalized COVID-19 patients found that tocilizumab did not decrease mortality or the progression to mechanical ventilation when compared to placebo (HR 0.83; CI 95% 0.38 to 1.84, *p* = 0.64). Additionally, it did not prevent disease progression (HR 1.11; CI 95% 0.59 to 2.10, *p* = 0.73). Hydroxychloroquine was also studied as it showed possible effectiveness in lowering COVID-19 infection in vitro. Despite this, several randomized controlled trials demonstrated significant cardiac side effects with no prophylactic or clinical benefits [44,45,46,47,48]. Addition of the macrolide antibiotic azithromycin also did not show any further clinical benefits, with a case-control study showing magnified cardiac toxicity [49]. Without clear benefits and valid toxicities, hydroxychloroquine and chloroquine have garnered a recommendation from the IDSA to avoid the use of these agents in the treatment of COVID-19.

Dipyridamole has amassed some attention following a small case series that showed that this medication improved clinical outcomes and suppressed SARS-CoV-2 replication in vitro [22]. Several phase 2 clinical trial are currently ongoing (NCT04391179, NCT04424901). However, as its benefits have not yet been proven in vivo, its use cannot be recommended outside its intended indications or in the context of clinical trials.

Lopinavir/ritonavir is a protease inhibitor used in the treatment of HIV and has demonstrated in vitro inhibition of SARS-CoV-1 and MERS-CoV replication [50,51]. It has also been evaluated for use in clinical practice in patients with acute respiratory distress syndrome (ARDS) [52,53]. This combination, when used with interferon beta-1b, was given early light from an open-label phase 2 study of 127 patients. The study showed a hastened nasopharyngeal swab clearance (7 days in the intervention group compared to 12 days in the controlled group, HR 4.37; 95% CI 1.86–10.24, *p* = 0.001) [54]. Since these early promising findings, there have been three randomized controlled trials, all showing no additional benefits from use of this combination with or without interferon [55,56,57]. However, the usefulness of this retroviral combination, with and without interferon beta-1b and ribavirin, is continuing to be explored for the treatment of COVID-19 in specialized populations, such as those with cancer and/or immunosuppressed patients (NCT04455958). Lopinavir/ritonavir is not recommended for routine use in the therapy of COVID-19.

Additional agents include nitazoxanide, penciclovir, and nelfinavir. Nitazoxanide is an antiprotozoal and has been shown to inhibit SARS-CoV-2 replication in vitro. Early use of this medication has shown promising effects in reducing SARS-CoV-2 viral load, but this did not translate to improved symptomology reduction, biochemical improvement, or clinical outcome [58]. There are still several ongoing phase 2 clinical trials (NCT04561219, NCT04348409) and an ongoing phase 3 clinical trial (NCT004463264) to study the utility of nitazoxanide in the treatment of patients with COVID-19. Penciclovir and nelfinavir have theoretical inhibitory effects on SARS-CoV-2 [59]. Currently there are no registered clinical trials looking into these antiviral medications for the treatment of COVID-19.

## 4. Adjunctive Nutraceuticals for COVID-19

The term nutraceutical is a combined terminology for nutrition and pharmaceuticals that popularly reflects the food or its part that has medicinal or health benefits [60]. The well known examples of nutraceuticals are milk, different vegetables, fruit juice, etc., as those food material also have health benefit roles [60,61]. Along with conventional treatment strategies, adjunctive use these nutraceuticals may also be beneficial in the treatment and/or prevention of COVID-19. Here, we have summarized several nutraceuticals that were evaluated for use in COVID-19.

### 4.1. Dietary Supplements

Dietary supplements are medications that are treated as food products by the FDA; therefore, unlike prescription medications, these medicines do not need to be proven safe or efficacious before marketing. Although not as rigorously evaluated, certain dietary supplements are still studied to assess their use in various disease states. Vitamin C, vitamin D, zinc, and melatonin are four dietary supplements that are currently being evaluated for use in COVID-19.

#### 4.1.1. Vitamins

Vitamins are micronutrients that perform an essential role in the proper structuring and functioning of proteins as well as in several physiological processes and signaling pathways in the body [62]. Vitamin C or ascorbic acid is a water-soluble vitamin that is proposed to have antioxidant, anti-inflammatory, and immunomodulatory properties [63,64,65,66]. Additionally, it serves as an enzyme cofactor for a number of biosynthetic processes and may increase the endogenous synthesis of catecholamines. Vitamin C has historically been studied for use in a number of viral and nonviral conditions, such as herpes zoster infection, influenza infection, and the common cold [67,68,69]. However, due to conflicting results and limited high-quality studies, the use of vitamin C for these indications is not routinely recommended.

There are several studies ongoing to evaluate the use of vitamin C in the treatment of COVID-19; however, results are not expected to be published until later in the year. A randomized controlled trial by Peng et al. hopes to reveal more evidence regarding the use of high doses of intravenous (IV) vitamin C (24 gm/day) and its effects on ventilator-free days and 28-day mortality [70]. Although not directly related to COVID-19, vitamin C has also been evaluated for use in septic shock and ARDS, both of which may be sequelae of COVID-19 infection; however, studies have found conflicting results for these indications. For example, a case series of 47 patients by Marik et al. found improved in-hospital mortality with the use of HAT therapy (IV hydrocortisone (50 mg q6h), vitamin C (1.5 g q6h), and thiamine (200 mg q12h)); however, a large, randomized controlled trial by Fujii et al. found no statistically significant difference in 90-day mortality between those who did and did not receive HAT therapy (28.6% vs. 24.5%, HR 1.18, 95% CI 0.69–2.00) [71,72]. Additionally, in a study by Fowler et al. of 167 patients with sepsis and ARDS, compared with those who had received placebo, a statistically significant difference in the change in the modified Sequential Organ Failure Assessment (SOFA) score was not found versus those who received high dose IV vitamin C (50 mg/kg), (3 point difference in the intervention group vs. 3 point difference in the placebo group, *p* = 0.86) [72]. A statistically significant difference was identified in 28-day mortality though (29.8% vs. 46.3%, *p* = 0.03). Notedly, mortality was evaluated at different time points in these studies.

Patients who are critically ill may also be deficient in vitamin C. Multiple studies found that a majority of septic patients have hypovitaminosis C [64,73,74]. As vitamin C cannot be endogenously produced, critically ill patients may require exogenous supplementation with vitamin C. As vitamin C has saturable and dose-dependent enteral absorption, IV administration is preferred if utilized in critically ill patients [64]. Although adverse effects are not common at over-the counter (OTC) dosages, at the high doses that have been studied for septic shock and ARDS, potential adverse effects include nausea, abdominal pain, calcium oxalate nephropathy, and glucose-6-phosphate dehydrogenase deficiency-induced hemolysis [64]. Point-of-care blood glucose measurements may also be inaccurate as vitamin C and glucose are structurally similar.

Vitamins D and E are also being evaluated as these fat-soluble vitamins may also have antioxidant and anti-inflammatory properties [73,74]. Of these two, vitamin D supplementation is of interest as low vitamin D levels may be correlated with an increased risk of ARDS and infectious diseases, including upper respiratory tract infections [75,76]. Additionally, a meta-analysis of 25 randomized controlled trials found that vitamin D supplementation reduced the risk of respiratory infection (adjusted OR 0.88, 95% CI 0.81–0.96, *p* < 0.001); however, various dosing strategies were utilized in the studies [77]. Vitamin D may also modulate adaptive immunity, facilitate the production of antibiotic peptides in the lungs to help prevent respiratory infection, and promote expression of the ACE2 gene [75,78]. As the ACE2 gene is downregulated by COVID-19, the use of vitamin D may help to balance the regulation of the renin–angiotensin–aldosterone system. Vitamin D may also attenuate the cytokine storm [79]. Although adverse effects are not common, toxicities associated with vitamin D may include nausea, confusion, hypercalcemia, abdominal pain, polyuria, and dehydration [80].

Circumstantial epidemiological and geographic evidence is also pointing toward a potential benefit of vitamin D as areas typically deficient in vitamin D have been shown to have a higher rate of mortality due to COVID-19 [75]. For example, a study by Laird et al. found that based on published data, countries with lower rates of vitamin D deficiency such as Norway and Finland had correspondingly lower levels of mortality (*p* = 0.46).

Due to factors such as the heterogeneity within the studies and the lack of high-quality evidence, it is difficult to provide exact recommendations for the use vitamins C, D, and E in this patient population; therefore, patients should receive at minimum their daily recommended vitamin intake. However, based on data from observational studies, a recent review proposed that patients at risk for COVID-19 take 10,000 IU/day of cholecalciferol for a few weeks, with a goal to raise 25-hydroxyvitamin D concentrations by at least 40–60 ng/mL (100–150 mmol/L) [78]. An ongoing study at the Queen Mary University of London, the COVIDENCE UK Study, hopes to reveal more evidence regarding the use of vitamin D, among other diet and lifestyle style modifications in COVID-19 (NCT04330599).

#### 4.1.2. Zinc

Zinc is an essential dietary trace element and the second most abundant trace element. It is vital to the preservation of both innate and acquired immunity as it is involved in the development, differentiation, and function of immune cells [81]. Although studies have been limited due to conflicting results regarding the exact benefit of its supplementation, zinc is commonly marketed for its use in upper respiratory tract viral infections such as the common cold. Zinc is believed to have antiviral effects due to its upregulation of tumor necrosis factor-alpha (TNF-α) and TNF-gamma and through its participation in metallothioneins, which may trigger apoptosis or decrease protein synthesis [81,82,83]. Zinc may also inhibit attachment to the nasopharyngeal mucous as well as viral replication. In vitro studies have shown that zinc may inhibit the template binding and elongation of SARS-CoV-1 RNA-dependent RNA polymerase (RdRp) to help prevent viral replication [84]. In vitro studies have also shown that due to its positive charge, chloroquine may act as an ionophone for zinc; therefore, numerous implications have been made regarding the potential synergy between chloroquine/hydroxychloroquine and zinc [82,85]. In one small case series of four patients, use of zinc salts were found to lead to a signification reduction in COVID-19 symptoms after invention initiation; however, a control was not used. Patients self-administered high doses of zinc salts, close to 200 mg daily via oral lozenges. Based on this report, the authors concluded that high dose supplemental zinc may have a role in the management of COVID-19 [86]. Another study showed COVID-19 patients with zinc deficiency (74.5 ug/dL vs. 105.8 ug/dL, *p* < 0.001) tended to require more corticosteroids (OR 7.2; CI 95% 1.39–37.35, *p* = 0.02) as well as longer hospital lengths-of-stay (OR 3.39; CI 95%, 0.99–11.57, *p* = 0.076) [87].

#### 4.1.3. Melatonin

Melatonin (N-acetyl-5-methoxytryptamine) may also be beneficial in the management of COVID-19 symptoms due to its anti-inflammatory, anti-oxidative, and immunomodulatory properties [88,89]. In respiratory syncytial virus models, melatonin has been shown to downregulate acute lung oxidative injury and inflammation [88]. Anti-inflammatory effects are thought to be through sirtuin-1 (SIRT-1) mediated downregulation of macrophage polarization and suppression of nuclear factor kappa-B (NF-κB). Melatonin also possesses immunomodulatory effects by enhancing the proliferation and maturation of lymphocytes. Although there are no current studies regarding the use of melatonin in COVID-19, future studies may provide further evidence regarding its use. Potential adverse effects of melatonin include dizziness, headache, nausea, and sleepiness; however, short-term use of melatonin, even at high doses, has been reported to be safe.

Due to the paucity of data and conflicting evidence, the use of dietary supplements such as vitamin C, vitamin D, zinc, and melatonin, cannot be justified in patients with COVID-19; however, as these medications are relatively well tolerated, their use may be considered based on individual patient needs. Dosing regimens are also difficult to discern due to the heterogeneity of doses that have been evaluated. Future studies hope to augment current evidence regarding the use of these medications in COVID-19. If oral dietary supplements are used, it is recommended to purchase supplements from United States Pharmacopeia (USP)-certified or verified brands to ensure the supplements contain the ingredients on the label and are manufactured with Good Manufacturing Practices (GMP) [90].

#### 4.1.4. Other Phytochemicals and Functional Foods

Phytochemicals or “plant” chemicals refer to a large variety of compounds, including carbohydrates, lipids, phenolics, terpenoids, alkaloids, and other nitrogen-containing compounds [91]. More recently, phytochemicals have been defined as chemicals from plants that do not meet the classical definition of “essential nutrients.” These compounds can be extracted for use or consumed via functional foods. Functional foods are merely the foods or herbs that naturally contain these biologically active ingredients [92]. For example, the Mediterranean diet (MD), commonly recommended for those with inflammatory diseases, consists of numerous functional foods that contain polyphenols, terpenoids, flavonoids, alkaloids, sterols, pigments, and unsaturated fats. Other well known examples of functional foods are milk, different vegetables, fruit juice, honey, etc. [60,61].

As with the aforementioned dietary supplements, many phytochemicals may also possess anti-inflammatory, immunomodulatory, and antiviral properties. In fact, a number of these phytochemicals have been previously evaluated for use for viruses within the coronavirus family and may prove beneficial for study with COVID-19. These phytochemicals are summarized in Table 2. The phytochemical that has been studied the most was glycyrrhizin, a triterpene saponin extracted from licorice plants [50,93,94]. In vitro studies found a selectivity index (SI) of approximately >67 with the use of glycyrrhizin; however, the most potent phytochemical evaluated appeared to be lycorine (SI = 954) [95]. A common target for a number of these antiviral phytochemicals was the 3Clike-protease (3CLpro). 3CLpro is the main protease that plays an important part in viral replication for coronaviruses. Phytochemicals such as sinigrin, aloe-emodin (366 µM), and hesperetin from *Isatis indigotica* root extracts attenuated the 3CLpro cleavage activity in a cell-based assay [96]. Amentoflavone from *Torreya nucifera*, eucalyptus isolated from *Lonicera japonica*, ginsenoside-Rb1 from *Panax ginseng*, and aqueous extract of *Houttuynia cordata* [97,98,99] are also associated with the downregulation of 3CLpro activity. Aescin, an important component of *Aesculus hippocastanum,* RH121 from Chinese Rhubarb (*Rheum palmatum*), flavonoids like herbacetin, rhoifolin, and pectolinarin from *Litchi chinensis*, and other similar phytochemicals such as quercetin, isobavaschalcone, 3-β-D-glucoside, and helichrysetin also inhibit 3CL proactivity of SARS-CoV [99,100,101,102]. Notedly, studies were limited by their in vitro and heterogenous designs; therefore, the clinical utility for these phytochemicals is not yet known.

Flavonoids from *Camellia sinensis*, extract of *Dioscoreae rhizoma*, *Taxillus chinensis*, and *Cibotium barometz* were also reported to play an important role in inhibiting the SARS-CoV 3CL protease activity and may possess potential antiviral activities against SARS-CoV [104,107,114]. Artimisinin from *Artemisia annua,* extract of *Pyrrosia lingua*, and *Lindera aggregata* inhibited the SARS-CoV-induced cytopathic effects in vero cells [95]. Medicinal plants such as *Stephania tetrandra*, *Scutettaria baicalensis*, and *Toona sinensis* were also reported to inhibit SARS-CoV viral replication [39,103,108]. Myricetin from *Myrica nagi* and scutellarin from *Scutellaria barbata* were reported to inhibit SARS-CoV helicase that ultimately lowered the ATPase activity, which may be responsible for their potential antiviral effects against SARS-CoV [108]. Kaempferol and their derivatives, which are present in several pear plants including *Opuntia cactus*, juglans from *Juglans regia*, emodin from *Cassia tora*, or its extract itself, were suggested to inhibit the 3a-channel protein of SARS-CoV for their antiviral effects [107,115]. Similar to bamlamivimab, tetra-O-galloyl-β-D-glucose and luteolin from *Galla chinensis* and *Veronicalina riifolia*, respectively, were reported to inhibit the binding of the SARS-CoV spike protein that prevents the entry of the virus into the host cell [110]. Curcumin from *Curcuma longa*, oleanane triterpenes from *Camellia japonica*, and extract of *Gentiana scabra* were also found to lower the replication and proliferation of SARS-CoV [107,112,113].

Routine use of these phytochemicals cannot be recommended outside of research as further studies are necessary to elucidate their potential benefit in the treatment and prevention of COVID-19. Several functional foods were also evaluated for use with coronaviruses as well as other respiratory viruses [116]. Examples of these include glycyrrhizin isolated from *Glycyrrhiza glabra,* tannic acid, 3-isotheaflavin-3-gallate (TF2B) from *C. sinensis* [93,117]. Ginger (*Zingiber officinale*) is also reported to have anti-inflammatory and antiviral effects [118]. In an in vitro study utilizing fresh and dried ginger, fresh ginger at a concentration of 300 μg/mL was found to decrease human respiratory syncytial virus plaque formation by 12.9%. Dried ginger was not able to inhibit viral plaque formation. Ginger is thought to be able to prevent early viral replication via blocking viral attachment and penetration into the cells. These phytochemicals need to be validated through further studies of their clinical effects in COVID-19 patients, despite having promising roles in experimental preclinical studies.

Honey is a functional food that is believed to contain antiviral, antibacterial, antioxidant, and anti-inflammatory properties [119]. This is because, in addition to sugars, honey contains amino acids, enzymes, vitamins, minerals, phenolic acids, and flavonoids. An in vitro study utilizing influenza-infected Madine-Darby canine kidney (MDCK) cells found that Manuka (*Leptospermum scoparium*) honey improved cell survival and inhibited viral replication. The exhibited 50% inhibitory concentration (IC50) for Manuka honey was 3.6 ± 1.2 mg/mL, with an SI of 22.9. Of interest, the SI for the control (zanamivir) was > 3.53 × 103. Additionally, when utilized in conjunction with neuraminidase inhibitors, the IC50′s of zanamivir and oseltamivir were reduced. Other studied functional foods for the influenza virus include several berries (raspberry, strawberry, lingonberry, and bilberry), soy proteins, and broccoli sprouts [120,121,122]. Broccoli sprouts are rich in precursors for sulforaphane antioxidants, which may affect natural killer cells to downregulate the influenza virus RNA level and lower the infection [122].

Similar to the dietary supplements mentioned previously, routine use of these phytochemicals and herbal extracts cannot be recommended due to the lack of high-quality evidence regarding their use in viral infections and COVID-19. For example, the majority of studies were conducted in vitro, with minimal agents having follow-up studies. Further studies are needed prior to recommending the routine use of these agents. Additional studies are also required prior to recommending the use of functional foods; however, risks may be minimal in adding standard quantities of such foods into one’s diet. Ideal amounts to consume of these functional foods are also difficult to discern as quantities of bioactive phytochemicals in the foods may vary from food products to food products [119].

## 5. Conclusions

**Scheme 1:**
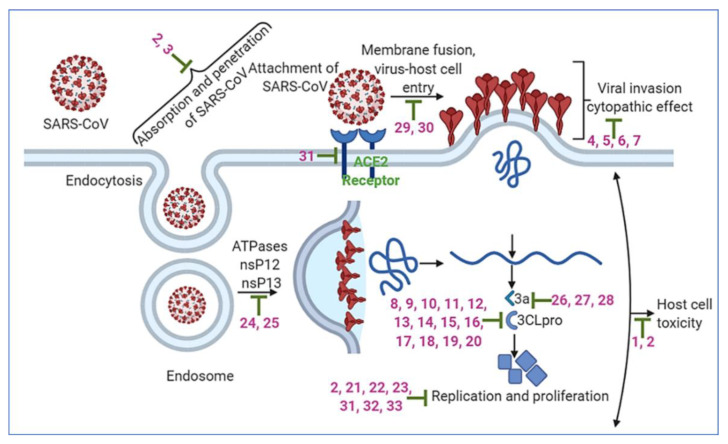
Mechanism targeted by supplements and nutraceuticals to lower the SARS-CoV2 mediated complication. 1. 3β-friedelanol, 3β-acetoxy friedelane, friedelin, and epitaraxerol from *Euphorbia neriifolia*; 2. Glycyrrhizin from licorice roots; 3. Saikosaponins A, B2, C, and D from *Radix bupleuri*; 4. Lycorie from *Lycoris radiata* extract; 5. Aritimisinin from *Artemisia annua*; 6. *Pyrrosia lingua* leaf extract; 7. Root extract of *Lindera aggregata*; 8. Sinigrin, beta-sitosterol, aloe-emodin, and hesperetin from *Isatis indigotica*; 9. Amentoflavone and other flavonoids from *Torreya nucifera*; 10. Extract of *Houttuynia cordata*; 11.Eucalyptus *Lonicera japonica*; 12. Ginsenoside-Rb1 from *Panax ginseng*; 13. Reserpine from *Rauwolfia serpentine*; 14. Aescin from *Aesculus hippocastanum*; 15. RH121 from *Rheum palmatum*; 16. Herbacetin, rhoifolin, and pectolinarin from *Litchi chinensis*; 17. Quercetin, epigallocatechin gallate, and gallocatechin gallate (GCG) from *Camellia sinensis*; 18. Rhizoma extract of *Dioscorea batatas*; 19. Extract of *Taxillus chinensis*; 20. Extract of *Cibotium barometz*; 21. tetrandrine (TET), fangchinoline (FAN), and cepharanthine (CEP) from *Stephania tetrandra*; 22. Baicalin and baicalein from *Scutellaria baicalensis*; 23. Bioactive fractions of *Toona sinensis*, 24. Myricetin from *Myrica nagi*; 25. Scutellarein from *Scutellaria barbata*; 26. Kaemferol from *Opuntia cactus*; 27. Juglanin from *Juglans regia*; 28. Emodin from *Cassia tora*; 29. Tetra-O-galloyl-β-D-glucose from *Galla chinensis*; 30. Luteolin from *Veronicalina riifolia*; 31. Curcumin from *Curcuma longa*; 32. Extract of *Gentiana scabra*; 33. Oleanane from *Camellia japonica*.

Due to the urgency revolving around COVID-19, numerous pharmaceuticals and nutraceuticals (as depicted in Scheme 1) are being evaluated for use [116] (e.g., NCT04395768, NCT04315948, NCT04401150, NCT04472585, NCT04411446, NCT04347382). As vaccinations are underway and we continue to pursue therapeutic strategies, there may be value in continuing to evaluate nutraceuticals as an additional area of study in the prophylaxis and treatment of COVID-19. Several nutraceuticals have already been shown to possibly have activity against viruses, including other species of coronavirus. However, due to the current lack of robust evidence regarding the safety and efficacy of these nutraceuticals, routine use cannot be recommended with confidence. In addition to pharmacological therapies, nonpharmacologic therapies such as physical distancing, quarantine, and infection-control should continue to be utilized as they are proven methods in preventing viral spread [123].

## Figures and Tables

**Table 1:** Conventional medications are currently being evaluated for use in COVID-19 infections.

Medication	Proposed Mechanism	Clinical Trial	Reference
Remdesvir (GS-5734)	Nucleotide analog that may inhibit virus replication	Phase 3, NCT04292730	[7,15]
Bamlamivimab	IgG1 monoclonal antibody against the spike protein of SARS-CoV-2, blocking the attachment to ACE2 receptors	Phase 2, NCT04427501	[16]
Dexamethasone	Potent corticosteroid with predominantly glucocorticoid activity Reduces the production of pro-inflammatory compounds	Phase 3, NCT04395105	[17,18]
Tocilizumab	Recombinant humanized monoclonal antibody against the IL-6 receptor Treatment of cytokine storm induced by SARS-CoV-2	Phase 3, NCT04356937: Phase 2, NCT04317092	[19]
Chloroquine/hydroxychloroquine	Prevents viral infection by blocking viral-cell fusion through alteration of the endosomal pH and glycosylation of cellular receptors Enhances host immune modulation	NCT04353271: Phase 3, NCT04447534	[15,20]
Azithromycin	Macrolide antibiotic that may have antiviral properties and immunomodulation properties, decreasing cytokine release	Phase 4, NCT04359316	[21]
Dipyridamole	Decreased SARS-CoV-2 replication in cells, clinical improvement potentially seen in cases of COVID-19 patients	Phase 2, NCT04391179: Phase 2, NCT04424901	[22]
Lopinavir/ritonavir	Protease inhibitor that may inhibit viral replication	Phase 2, NCT04455958	[7,23]
Ribavirin	Guanosine analog that inhibits viral RNA synthesis	Phase 2, NCT04494399	[15,24]
Nitazoxanide	Antiprotozoal and antiviral agent that inhibits the SARS-CoV-2	Phase 2, NCT04552483: Phase 2, NCT04561219: Phase 3, NCT04348409: Phase 3, NCT04463264	[15]
Baricitinib	Selective Janus kinase (JAK) 1 and 2 inhibitor	Phase 3, NCT04401579 Phase 3, NCT04421027	[25,26]
Penciclovir	Reduced SARS-CoV-2 replication	NA	[15]
Nelfinavir	Protease inhibitor that inhibits viral replication	NA	[27]

**Table 2:** Phytochemicals that may be beneficial as adjunct agents in COVID-19.

Plant	Phytochemicals	Proposed Mechanism	Outcomes	Reference
*Glycyrrhiza glabra; Glycyrrhiza uralensis* (licorice)	Glycyrrhizin (triterpenoid saponins)	Inhibits viral replication and adsorption/penetration via induction of nitrous oxide synthase	EC50 of 300 ± 51 mg/L and CC50 of >20,000 mg/L of SARS-CoV-infected Vero cells SI of >67	[50,93,94]
*Bupleurum* spp.; *Heteromorpha* spp.; *Scrophularia scordonia*	Saikosaponin B2	Interferes with early-stage viral replication	EC50 of 1.7 ± 0.1 μM/L and CC50 of 383.3 ± 0.2 μM/L of HCoV-229E-infected MRC5 cells SI of 221.9	[103]
*Stephania tetrandra*	TET FAN CEP	Inhibits viral replication as well as viral S and N protein expression to prevent viral entry	EC50 of 0.33 ± 0.03 µM and CC50 of 13.41 ± 0.36 µM for TET in HCoV-OC43-infected MRC-5 cells SI of 40.2, 11.45 13.6 for TET, FAN, and CEP, respectively	[101]
*Isatis indigotica*	Indigo Sinigrin Hesperetin	Inhibits viral replication by blocking the cleavage of the 3CL^pro^ of SARS-CoV	IC50 for hesperetin and sinigrin was 8.3 µM and 217 µM, respectively	[96]
*Torreya nucifera*	Amentoflavone	Inhibits viral replication by noncompetitively blocking cleavage of the 3CL^pro^ of SARS-CoV	IC50 of 8.3 ± 1.2 µM	[97]
Flaxseed	Herbacetin	Inhibits viral replication by blocking the cleavage of the 3CL^pro^ of SARS-CoV	A flavonoid found to have an IC50 of 33.17 µM	[104,105]
*Rhus succedanea*	Rhoifolin	Inhibits viral replication by blocking the cleavage of the 3CL^pro^ of SARS-CoV	A flavonoid found to have an IC50 of 27.45 µM	[104]
*Cirsium chanreoenium*	Pectolinarin	Inhibits viral replication by blocking cleavage of the 3CL^pro^ of SARS-CoV	A flavonoid found to have an IC50 of 37.78 µM	[104,106]
*Houttuynia cordata*	-	Inhibits viral replication via an effect on 3CL^pro^ and through immunostimulatory effects	Dose-dependent inhibition of 3CL^pro^ activity Stimulated proliferation of CD4 and CD8 T cells	[98]
*Rheum palmatum L.* (Chinese rhubarb)	-	Inhibits viral replication by blocking the cleavage of the 3CL^pro^ and also interferes with the interaction of SARS-CoV S protein and ACE2	From the anthraquinones extracts, the IC50 ranged from 13.76 ± 0.03 to 59.33 ± 6.52	[100]
*Cibotium barometz*	Rhizoma cibotii	Inhibits viral cytopathogenic effect and inhibits viral replication via effects on 3CL^pro^	CBE extract was found to have an EC50 of 8.42 mcg/mL and CC50 > 500 μg/mL in SARS-CoV-infected Vero E6 cells SI of >59.4	[107]
*Gentiana scabra*	Gentianae radix	Inhibits viral cytopathogenic effect and inhibits viral replication via effects on 3CL^pro^	EC50 of 8.7 mcg/mL and CC50 >500 μg/mL in SARS-CoV-infected Vero E6 cells SI of >57.5	[107]
*Dioscorea batatas*	Discoreae rhizome	Inhibits viral cytopathogenic effect and inhibits viral replication via effects on 3CL^pro^	EC50 8.06 μg/mL and CC50 > 500 μg/mL in SARS-CoV-infected Vero E6 cells SI of >62	[107]
*Cassia tora*	Cassiae semen	Inhibits viral cytopathogenic effect and inhibits viral replication via effects on 3CL^pro^	EC50 of 8.43 μg/mL and CC50 > 500 μg/mL in SARS-CoV-infected Vero E6 cells SI of >59.3	[107]
*Taxillus chinensis*	Loranthani rhamus	Inhibits viral cytopathogenic effect and inhibits viral replication via effects on 3CL^pro^	EC50 5.39 μg/mL and CC50 > 500 μg/mL in SARS-CoV-infected Vero E6 cells SI of >92.8	[107]
*Ceratonia siliqua*	Myricetin	Inhibits viral replication by blocking the ATPase activity of the SARS-CoV helicase protein nsP14; may also have antioxidant properties	Inhibited ATPase activity by > 90% at a concentration of 10 µM	[108]
*Scutettaria baicalensis*	Secutellarein	Inhibits viral replication by blocking the ATPase activity of the SARS-CoV helicase protein nsP14	Inhibited ATPase activity by > 90% at a concentration of 10 µM	[108]
*Aesculus hippocastanum* (horse chestnut tree)	Aescin	Inhibition of viral replication via unknown mechanism; may also have anti-inflammatory properties	EC50 of 6 µM and CC50 of 15 µM SI of 2.5	[99]
*Rauwolfia serpentine*	Reseprine	Inhibition of viral replication via an unknown mechanism	EC50 of 3.4 µM and CC50 of 25 µM SI of 7.3	[99]
*Juglans regia*	Juglanin	Inhibits interaction between virus and host cells via blocking the 3a-protein channel	IC50 of 2.3 µM	[109]
*Galla chinensis*	TGG	Interferes with viral cell fusion via effects on the S protein	EC50 of 4.5 (1.96–5.8) µM and CC50 of 1.08 mM in SARS-CoV-infected Vero E6 cells SI of 240	[110]
*Veronicalina riifolia*	Luteolin	Interferes with viral cell fusion via effects on the S protein	EC50 of 10.6 (9.2–12.2) µM and CC50 of 0.155 mM in SARS-CoV-infected Vero E6 cells SI of 14.6	[110]
*Lycoris radiata*	Lycorine	Inhibits viral cytopathic effect via an unknown mechanism	EC50 of 15.7 ± 1.2 μg/mL and CC50 of 14,980 ± 912 μg/mL of SARS-CoV-infected Vero E6 cells SI of 954	[95]
*Euphorbia neriifolia L*	3β-friedelanol (triterpenoid)	Inhibits viral activity via an unknown mechanism	132.4% cell survival vs. 69.5% with the control of actinomycin D in HCoV-229E MRC5 cells	[111]
*Scutellaria baicalensis*	Baicalin	Inhibits viral activity via an unknown mechanism	EC50 of 12–50 μg/mL at 48–72 h in SARS-CoV-infected fRHK4 cells EC50 = 100 μg/mL at 48 h in SARS-CoV-infected Vero-E6 cells	[50]
*Panax ginseng*	Ginsenoside-Rb1	Inhibits viral activity via an unknown mechanism	MIC of 100 µM toward SARS-CoV	[112]
*Toona sinensis*	TSL-1	Inhibits viral activity via an unknown mechanism	EC50 of 30 μg/mL, when boiled and used with SARS-CoV-infected Vero cells SI of 17	[113]

Abbreviations: EC50 = effective concentration of compound needed to inhibit the cytopathic effect to 50% of control value; IC50 = inhibitory concentration of the compound to achieve 50% inhibition; CC50 = cytotoxic concentration of the compound that reduced cell viability to 50%; SI = selectivity index (=CC50/EC50); MIC = minimum inhibitory concentration; fRHK-4 = fetal rhesus kidney-4; 3CLpro = 3C-like proteases; TET = bis-benzylisoquinoline alkaloids tetrandrine; FAN = fangchinoline; CEP = cephrananthine.

## Data Availability

All the data are presented within this article.
